# Fulminant Neurologic Manifestation of Sjogren’s Syndrome: A Case Report

**DOI:** 10.7759/cureus.42604

**Published:** 2023-07-28

**Authors:** Rosy M Laxmidhar, Fehmida Laxmidhar, Kavit Shastri, Sahil Patel, Shivani Patel

**Affiliations:** 1 Internal Medicine, Byramjee Jeejeebhoy (BJ) Medical College, Civil Hospital Asarwa, Ahmedabad, IND; 2 Internal Medicine, Western Reserve Health/Northeast Ohio Medical University (NEOMED), Warren, USA

**Keywords:** potassium balance, metabolic acidosis, renal tubular acidosis, hypokalemic paralysis, sjogren's syndrome

## Abstract

Sjogren's syndrome (SS) is an autoimmune disorder characterized by the destruction of exocrine glands by lymphocytic infiltration. Children and teenagers are less commonly affected. The initial symptoms of SS in teenagers might vary, depending on whether parotitis or other systemic organ involvement is present. Glandular involvement with the clinical hallmarks of dry eyes and dry mouth is common. Our case report is about a young woman who was admitted with acute flaccid paralysis and severe respiratory distress with extremely low serum potassium; further investigation revealed distal renal tubular acidosis. The patient was ultimately diagnosed with primary SS with high levels of SS-a/SS-b antibodies and a positive Schirmer's test. Our case demonstrates that hypokalemic paralysis can be a manifestation of SS, even though it is a rare cause.

## Introduction

Sjogren's syndrome (SS) is a chronic inflammatory systemic autoimmune illness that causes lacrimal and salivary glands to gradually degenerate due to lymphocytic infiltration [1]. SS may result in renal complications even before the onset of sicca symptoms. The prevalent renal manifestation of SS is chronic interstitial nephritis. The symptoms of this condition vary and may include Fanconi syndrome, distal renal tubular acidosis (dRTA), nephrogenic diabetes insipidus, or mild asymptomatic hypokalemia [2]. Patients of dRTA frequently show up with few or no symptoms, which might cause a delay in diagnosis. Progressively, it can cause serious acid-base imbalances, such as hyperchloremic metabolic acidosis and fatally low potassium levels [3]. Our patient developed quadriplegia with severe hypokalemia. Detailed history, clinical examination, and laboratory assessment of the patient narrowed down the diagnosis to normal anion gap metabolic acidosis with distal RTA as the cause. Keeping in mind the gender of the patient and mild sicca symptoms, we performed additional tests and finally figured out primary SS as the root cause of the problem. Even though RTA leading to hypokalemic paralysis is not common, we emphasize that hypokalemic paralysis can be the first sign of SS in a patient. The manifestation of hypokalemia can precede the usual glandular symptoms and bring to light an undetected SS.

## Case presentation

An 18-year-old female presented to the emergency department with complaints of acute onset, progressive quadriplegia followed by difficulty breathing. All four limbs were involved simultaneously. There was no complaint of abdomen pain, vomiting, diarrhea, or fever. The patient denied having diplopia, dysphagia, diurnal variation of symptoms, or slurring of speech. She did not experience a similar episode before. There was no history of drugs, canned food intake, or alteration in bowel and bladder movements. Her history was unremarkable for diabetes, hypertension, and tuberculosis. Her family history was insignificant for such a presentation. Based on the history provided, we could rule out Guillain-Barre syndrome, myasthenia gravis, spinal cord injury, botulism, and stroke as potential causes of paralysis. She was examined in a well-lit room and was conscious and oriented to time, place, and person. Her blood pressure was 110/70 mmHg, heart rate 80/min, respiratory rate 46/min, and single breath count of 6. Examination of her nervous system revealed acute, flaccid paralysis of all four limbs with reduced tone, muscle strength of 2/5, and bilateral areflexia. There was no involvement of cranial nerves, normal higher motor functions, and absence of sensory deficit. Her blood work revealed extremely low serum potassium (1.68 mmol/L). The venous blood gas analysis results and subsequent tests are shown in Table 1. U waves, ST segment depression, and shallow T waves were visible on the electrocardiogram. Abdominal X-ray indicated stones in the left ureter for which the patient was asymptomatic. The patient's cardiopulmonary assessment was normal, and no organomegaly was palpated.

**Table 1:** Laboratory parameters pCO2: partial pressure of carbon dioxide; pO2: partial pressure of oxygen; HCO3: bicarbonate; Hb: hemoglobin; MCV: mean corpuscular volume; HIV: human immunodeficiency virus; HbsAg: hepatitis B surface antigen; HCV: hepatitis C virus; SSA: Sjögren's syndrome-related antigen A; SSB: Sjögren's syndrome-related antigen B; TSH: thyroid stimulating hormone; ALT: alanine transaminase; ALP: alkaline phosphatase.

Test	Value
Venous blood gas analysis	
pH	7.14
pCO2	42.2 mmHg
pO2	33.2 mmHg
HCO_3_	14.2 mmol/L
Oxygen saturation	48%
Serum osmolality	304 mOsm/kg
Serum electrolytes	
Sodium	147.3 mmol/L
Potassium	1.68 mmol/L
Chloride	127.2 mmol/L
Calcium	8.3 mg/dL
Urinalysis (sample: spot urine)	
pH	7.0
Urinary potassium	10.3 mEq/L
Urinary sodium	22.0 mEq/L
Urinary chloride	20.0 mEq/L
Urine anion gap	12.3 (positive)
Urine glucose	negative
Urine osmolality	330 mOsm/kg
Urine creatinine	16 mg/dL
Urine potassium-to-creatinine ratio	64.37 mmol/g
Trans-tubular potassium gradient	6
Complete blood count	
Hb	9.2 g/dL
MCV	72 fl
Total leukocyte count	12.26 × 10^3^/cumm
Platelets	401 × 10^3^/cumm
Viral markers	
HIV	negative
HBsAg	negative
Anti-HCV	negative
Autoimmune profile	
Anti-SSA	100 U/mL (reference: 12.0 U/mL)
Anti-SSB	64 U/mL (reference: 12.0 U/mL)
Serum TSH	3.05 uIU/ml
Kidney function tests	
Urea	43 mg/dL
Creatinine	0.84 mg/dL
Liver function tests	
ALT	30 IU/L
ALP	82 U/L
Total protein/albumin	9.1/4.43 g/dL

**Figure 1:**
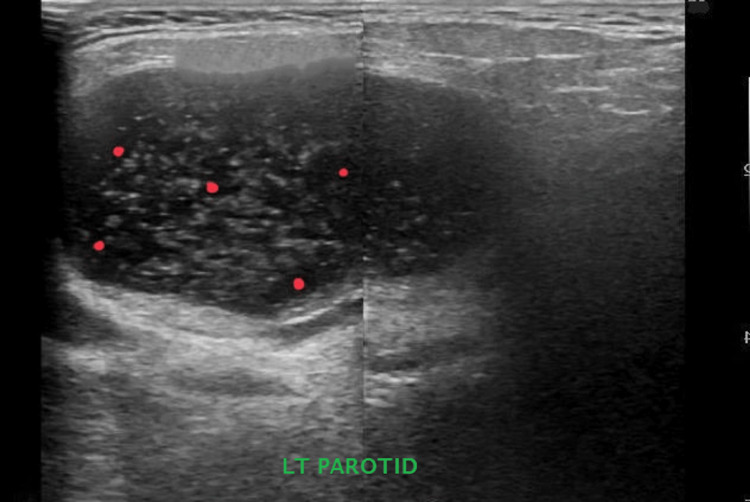
Ultrasound of the left parotid gland showing the presence of multiple variable-sized cystic areas (red dots) in the superficial and deep lobes of the gland, with internal striations favoring benign lymphoepithelial lesions of the left parotid gland. LT: left

The laboratory parameters like normal anion gap metabolic acidosis, positive urine anion gap, urine pH 7, and absence of glucose in urine aroused high suspicion for distal RTA as the probable cause. The patient developed increasing shortness of breath and was electively intubated due to respiratory muscle weakness. To determine the cause of RTA, additional testing was planned. Medullary sponge kidney and obstructive uropathy were ruled out as potential causes of dRTA due to the normal ultrasound of the abdomen. The thyroid functions were within normal limits, calcium levels within the reference range, and the individual did not test positive for viral markers of hepatitis B, hepatitis C, and HIV. On further interrogation, the patient admitted having a dry mouth and a gritty sensation in the eyes. Schirmer's test showed a positive result (Schirmer-1: 3 mm in the right eye/2 mm in the left eye; Schirmer-2: 3 mm in the right eye/1 mm in the left eye). Her autoantibody profile was favorable for anti-RO/ SS-related antigen A (SSA) and anti-La/SS-related antigen B (SSB) (Table 1). Ultrasound of the left parotid gland showed the presence of multiple variable-sized cystic areas in the superficial and deep lobes of the gland, with internal striations favoring benign lymphoepithelial lesions of the left parotid gland (Figure 1). These findings fulfilled the 2016 American College of Rheumatology (ACR)/the European League Against Rheumatism (EULAR) classification criteria for primary SS. The conclusive diagnosis was SS-induced distal RTA leading to hypokalemic paralysis.

The patient underwent intensive treatment with 40 mEq/hour of potassium chloride via a central line and oral bicarbonate to maintain the ongoing renal loss. On the second day of admission, she was extubated after a successful spontaneous breathing trial. After four days of hospitalization, the patient's potassium levels remained stable at 3.5-3.9 mEq/dl, and her symptoms were mild. She was discharged with advice regarding diet, regular monitoring, and physiotherapy. She was referred to the rheumatology department for further evaluation. 

## Discussion

SS encompasses various diseases, ranging from dry eyes and mouth to systemic involvement and symptoms outside the glands. Additionally, SS can be linked to the development of cancers, specifically non-Hodgkin's lymphoma. The prevalence of SS is higher among middle-aged females and less frequent among children and adolescents. However, the diagnosis is often overlooked in some cases, particularly when severe sicca symptoms are absent, as observed in our patient. A study conducted by Goules et al. [4] provides a comprehensive summary of different clinical phenotypes of SS that deserve clinical attention. Younger study participants exhibited higher rates of B cell-related manifestations, including salivary gland enlargement, hypergammaglobulinemia, presence of autoantibodies, leukopenia, C4 hypocomplementemia, and lymphoma. On the other hand, older participants had higher rates of dry mouth, interstitial lung disease, and lymphoma.

The prevalence of complete dRTA in patients with primary SS is reported to be 5%, as indicated in the studies by Tim et al. [5] and Bosseni et al. [6]. dRTA is characterized by renal tubular dysfunction, leading to an inability to maintain physiologic acid-base balance properly. The hallmark of dRTA is the failure of the distal portions of the nephron to acidify the urine.

When patients present signs of severe potassium deficiency, analyzing their acid-base levels can help narrow down potential causes, such as dRTA. The exact mechanism by which SS can lead to dRTA remains uncertain. However, earlier research suggested that in SS patients, the vacuolar H+-ATPase was downregulated in the A-intercalated cells, while AE1 (pendrin) was concurrently underexpressed in the B-intercalated cells. It is believed that the significant malfunction in SS is caused by reduced secretion of H+ and that the deregulation of pendrin serves as a compensatory mechanism to lower HCO_3_- secretion and prevent further acidosis [7].

In autoimmune diseases like primary SS, distal RTA typically presents with mild or no symptoms. However, inadequate potassium levels in the blood can occasionally lead to unexpected paralysis and breathing difficulties, potentially revealing an undisclosed medical condition. A study by Ungureanu et al. [8] revealed that among 37 cases of dRTA-induced hypokalemic paralysis, only three progressed to respiratory failure, necessitating orotracheal intubation and ventilation. This highlights the rarity of such cases. Similar incidents have been documented in the literature as lone case reports [9, 10].

Thus, it is crucial to thoroughly assess cases of RTA to avoid adverse outcomes, detect a possibly manageable condition, and halt the development of chronic kidney disease. Additional diagnostic testing should be performed on adolescent patients with evidence of renal pathologies to rule out SS, as young individuals may exhibit various symptoms inconsistent with the disease's typical presentation. Moreover, there should be prompt management of metabolic disturbances with potassium and alkali therapy to avoid potentially fatal consequences.

## Conclusions

This case calls attention to including SS as a differential diagnosis and performing autoimmune investigations in any patient presenting with hypokalemic paralysis from dRTA, even in the absence of severe sicca symptoms. Although mild asymptomatic renal disease is frequent in SS, hypokalemic paralysis, and sometimes respiratory failure, can be the first signs of the condition. Early diagnosis and treatment can lead to an improved disease prognosis.
